# Multiplex detection of antibodies to Chikungunya, O’nyong-nyong, Zika, Dengue, West Nile and Usutu viruses in diverse non-human primate species from Cameroon and the Democratic Republic of Congo

**DOI:** 10.1371/journal.pntd.0009028

**Published:** 2021-01-21

**Authors:** Raisa Raulino, Guillaume Thaurignac, Christelle Butel, Christian Julian Villabona-Arenas, Thomas Foe, Severin Loul, Simon-Pierre Ndimbo-Kumugo, Placide Mbala-Kingebeni, Sheila Makiala-Mandanda, Steve Ahuka-Mundeke, Karen Kerkhof, Eric Delaporte, Kevin K. Ariën, Vincent Foulongne, Eitel Mpoudi Ngole, Martine Peeters, Ahidjo Ayouba

**Affiliations:** 1 Recherches Translationnelles sur le VIH et Maladies Infectieuses/INSERM U1175, Institut de Recherche pour le Développement et Université de Montpellier, France; 2 Centre de Recherches sur les Maladies Émergentes, Ré-émergentes et la Médecine Nucléaire, Institut de Recherches Médicales et D'études des Plantes Médicinales, Yaoundé, Cameroun; 3 Institut National de Recherche Biomédicales, Kinshasa, République Démocratique du Congo; 4 Department of Biomedical Sciences, Virology Unit, Institute of Tropical Medicine, Antwerp, Belgium; 5 Department of Biomedical Sciences, University of Antwerp, Antwerp, Belgium; 6 Département de bactériologie-virologie, CHU de Montpellier, Montpellier, France; Center for Disease Control and Prevention, UNITED STATES

## Abstract

**Background:**

Epidemic arbovirus transmission occurs among humans by mosquito bites and the sylvatic transmission cycles involving non-human primates (NHPs) still exists. However, limited data are available on the extent in NHPs infections and their role. In this study, we have developed and validated a high-throughput serological screening tool to study the circulation of multiple arboviruses that represent a significant threat to human health, in NHPs in Central Africa.

**Methodology/Principal findings:**

Recombinant proteins NS1, envelope domain-3 (DIII) for the dengue (DENV), yellow fever (YFV), usutu (USUV), west nile (WNV) and zika (ZIKV) and envelope 2 for the chikungunya (CHIKV) and o'nyong-nyong (ONNV) were coupled to Luminex beads to detect IgG directed against these viruses. Evaluation of test performance was made using 161 human sera of known arboviral status (66 negative and 95 positive). The sensitivity and specificity of each antigen were determined by statistical methods and ROC curves (except for ONNV and USUV). All NS1 antigens (except NS1-YFV), CHIKV-E2 and WNV-DIII had sensitivities and specificities > 95%. For the other DIII antigens, the sensitivity was low, limiting the interest of their use for seroprevalence studies. Few simultaneous reactions were observed between the CHIKV+ samples and the NS1 antigens to the non-CHIKV arboviruses. On the other hand, the DENV+ samples crossed-reacted with NS1 of all the DENV serotypes (1 to 4), as well as with ZIKV, USUV and to a lesser extent with YFV. A total of 3,518 samples of 29 species of NHPs from Cameroon and the Democratic Republic of Congo (DRC) were tested against NS1 (except YFV), E2 (CHIKV/ONNV) and DIII (WNV) antigens. In monkeys (n = 2,100), the global prevalence varied between 2 and 5% for the ten antigens tested. When we stratified by monkey’s biotope, the arboreal species showed the highest reactivity. In monkeys from Cameroon, the highest IgG prevalence were observed against ONNV-E2 and DENV2-NS1 with 3.95% and 3.40% respectively and in DRC, ONNV-E2 (6.63%) and WNV-NS1 (4.42%). Overall prevalence was low in apes (n = 1,418): ranging from 0% for USUV-NS1 to 2.6% for CHIKV-E2. However, a very large disparity was observed among collection site and ape species, *e*.*g*. 18% (9/40) and 8.2% (4/49) of gorillas were reactive with CHIKV-E2 or WNV-NS1, respectively in two different sites in Cameroon.

**Conclusions/Significance:**

We have developed a serological assay based on Luminex technology, with high specificity and sensitivity for simultaneous detection of antibodies to 10 antigens from 6 different arboviruses. This is the first study that evaluated on a large scale the presence of antibodies to arboviruses in NHPs to evaluate their role in sylvatic cycles. The overall low prevalence (<5%) in more than 3,500 NHPs samples from Cameroon and the DRC does not allow us to affirm that NHP are reservoirs, but rather, intermediate hosts of these viruses.

## Author summary

In the last decades, chikungunya, zika, yellow fever, usutu and dengue viruses have (re)-emerged in different parts of the world and many of these outbreaks occur in resource-limited countries with limited or under-equipped health facilities and where endemic malaria with very similar clinical symptoms confounds surveillance. Most arboviruses that circulate today likely originated in Africa where sporadic human outbreaks occur. In this work, we developed a serological tool that allows simultaneous detection of IgG antibodies to multiple arbovirus in a biological sample. With this highly sensitive and specific multiplex assay, we screened more than 3,500 samples collected from 29 species of monkeys and apes in Africa. We found a global IgG antibody prevalence of less than 5%. However, this seroprevalence varied by collection site, NPHs species and virus type. Given these findings, we concluded that African non-human primates are most likely not the reservoirs, but rather are intermediate hosts.

## Introduction

Ecology and human behavior play a major role in the increasing emergence and re-emergence of infectious diseases and several factors are required for this to occur [1]. These include prevalence of pathogens in the natural host, transmission mode of the pathogens, frequent contact between humans and wildlife, capacity to adapt to a new host and conditions for subsequent epidemic spread into the human population [2,3]. In particular, the increased contact between humans and wildlife lead to increased risk for disease emergence in humans [4,5].

In the last decades, chikungunya (CHIKV), zika (ZIKV), yellow fever (YFV), usutu (USUV) and dengue (DENV) viruses have (re)-emerged in different parts of the world [6–8], many of these outbreaks occur in resource-limited countries with limited or under-equipped health facilities and where endemic malaria with very similar clinical symptoms confounds surveillance [9]. Most arboviruses that circulate today likely originated in Africa where sporadic human outbreaks occur: YFV is known to circulate endemically in Sub-Saharan Africa for centuries [10]; west nile virus (WNV) and ZIKV were first identified in 1937 and 1947, respectively, in Uganda [11]; CHIKV in 1952 in Tanzania [12] USUV in 1959 in South Africa. The precise origins of DENV remain unknown but it is widely prevalent in East, Central and South Africa.

While large outbreaks of arbovirus diseases in human populations are well documented [13–15], many questions remain unanswered on their sylvatic-cycles, in particular, which wildlife species could be involved. In almost any review or research article, the zoonotic cycles for ZIKV, DENV, YFV CHIKV are presented with arboreal mosquitos feeding on non-human primates (NHPs) but this is based on very limited data. There is only very limited evidence of arbovirus infection or exposure in NHPs but also in wildlife in general. Studying the animal reservoir of arboviruses is a challenging question, because (*i*) there are difficulties inherent in wildlife sampling, (*ii*) there are limited high throughput screening technologies, (*iii*) the antigenic proximity among arboviruses hinders specificity and (*iv*) arboviruses cause acute infections which limits the detection of the virus to a very narrow window of time, rarely exceeding 3 weeks [16]. The detection of antibodies against arboviruses antigens represent an alternative to virus detection but information on seroprevalence in wildlife are very limited. A few studies reported prevalence of antibodies (mostly IgG) in different African non-human primates (NHPs) species (reviewed in Valentine and colleagues in 2019) [17], which varied from 0% to 100%, depending on the species, the targeted arbovirus, the detection method used, the country of sample’s origin and the number of samples tested.

In the present work, we have addressed some of these challenges. We first developed a high throughput serological screening tool based on the Luminex technology. Next, we screened more than 3,500 samples of a wide diversity of NHPs species from Cameroon and the Democratic Republic of Congo (DRC) for presence of antibodies to multiple arboviruses in order to evaluate their potential role in sylvatic cycles. Our data show an overall low seroprevalence of IgG antibodies to arboviruses unevenly distributed according to NHPs species and to sample collection site.

## Materials and methods

### Human panel samples and ethics statement

We used a panel of 161 samples of known arbovirus serostatus to validate our Luminex based serological test **(S1 Table)**. All the human samples used in this study were anonymized, and there is no way to link back these leftovers to the 161 patients. The panel consisted of 66 arbovirus negative leftover plasma samples from the Virology department of the University Hospital, Montpellier, France and the Institute of Tropical Medicine, Antwerp, Belgium. The patients were referred to these laboratories for various illnesses and were tested for the presence of an arboviral infection by IgM and IgG Immunofluorescence assay or ELISA assays, PCR and sero-neutralization for some of them. All 66 negative control samples used in the present study were negative with all these assays. Positive control samples for CHIKV, DENV, WNV, YFV and ZIKV originated from patients in France, Belgium, Colombia and the DRC. The samples were obtained as follows: during outbreaks (CHIKV and YFV in the DRC, DENV in Colombia and WNV in France); returning travelers in Europe from countries with outbreaks (ZIKV, DENV) or vaccinees (YFV in Colombia). DENV positive sera consisted of six DENV-1, seven DENV-2, four DENV-3, three DENV-4 and three DENV-1,2,3,4 reactive samples. Outbreak and returning travelers’ samples were confirmed positive by PCR and follow-up serum samples, confirmed by commercial serological assays, were used in the present study. For USUV, we had only three samples from experimentally infected mice that were serially bleeded. We were not able to get positive controls for o’nyong nyong virus (ONNV).

### Samples from monkeys and apes

**Fig 1:**
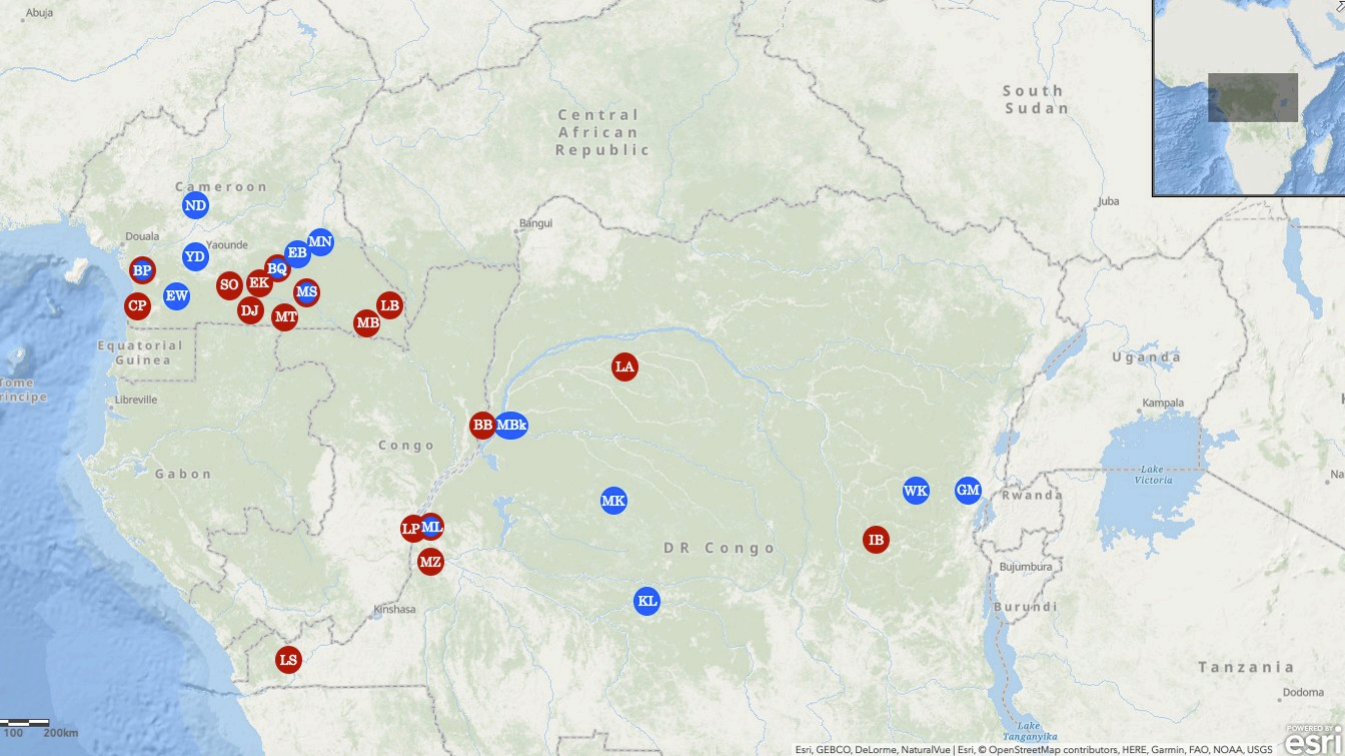
Sample collection sites. Sites where samples from non-human primates (NHPs) were collected are highlighted with circles on the maps, as follows: blue indicates sites where bushmeat samples from monkeys were collected; red, sites where fecal samples from apes were collected; blue and red, sites where bushmeat samples from monkeys and fecal samples from apes were collected. Abbreviations of sites are as follows: BP, Bipindi; BQ, north of Dja; EB, Eboumetoum; EW, Ebolowa; GM, Goma; KL, Kole; MBk, Mbandaka; MK, Monkoto; ML, Malebo; MN, Mindourou; MS, Messok; ND, Nditam; WK, Walikale; YD, Yaoundé.

We tested samples from bushmeat and pet monkeys from studies that were conducted between 1999 and 2016 on simian retroviruses and the origin of HIV [18–20] in 14 different sites in southern Cameroon and the DRC (**Fig 1** and **S2A Table**). Whole blood was collected from monkey bushmeat, either by intracardiac puncture and subsequent storage at –20°C, or as a dried blood spot (DBS) on Whatman 903 filter paper (GE Healthcare) at the points of hunting injury and spotting, as described previously [21]. Blood was drawn on EDTA tubes from pet monkeys by venipuncture after tranquilization with ketamine [20]. Species were visually identified in the field and confirmed on a subset of samples by 12S sequence analysis, as previously described [22]. Fecal samples were collected between 2005 and 2017 from wild common chimpanzees (*Pan troglodytes troglodytes*), western lowland gorillas (*Gorilla gorilla gorilla*), eastern lowland gorillas (*Gorilla beringei graueri***)** and bonobos (*Pan paniscus*) at 18 different sites in Cameroon and DRC as part of studies on the origin of HIV [23] (**Fig 1 and S2B Table).** Feces were stored in RNA-later (Sigma-Aldrich, Saint-Quentin Fallavier, France), kept at ambient temperature in the field for a maximum of three weeks, and then stored at –20°C or –80°C in central repository laboratory.

### Screening for IgG antibodies to arboviruses

#### Recombinant proteins

We used different commercially available recombinant proteins derived from the envelope or non-structural proteins of CHIKV, ONNV, DENV, ZIKV, USUV, YFV and WNV viruses **(S3 Table**). The proteins were purchased already purified (all above 95% of purity, except DENV4_NS1 and CHIKV_NSP at >90%) as lyophilized powders, and resuspended in a buffer at concentration as per manufacturer’s instructions, aliquoted and stored frozen at -20°C until use.

#### Protein coupling to Luminex beads

We used our previously described protocol for coupling primary amines bearing moieties (peptides and proteins) to Luminex beads [21,24]. Briefly, recombinant proteins (1–4μg/1.25 x10^6^ beads) were covalently coupled on carboxyl functionalized fluorescent magnetic beads (Luminex Corp., Austin, TX) with the BioPlex amine coupling kit (Bio-Rad Laboratories, Marnes-la-Coquette, France) according to the manufacturer’s instructions. We blocked unreacted sites with blocking buffer from the amine coupling kit. Protein-coupled microsphere preparations were washed with PBS, and stored in storage buffer (Bio-Rad) at 4°C in the dark until use.

#### Multiplex screening for IgG antibodies to arbovirus in plasma and fecal dialysates

Before use, recombinant protein-coupled beads were vortexed for 30s and diluted to 2,000 beads/μl of assay buffer (Phosphate Buffered Saline (PBS) containing 0.75 mol/L NaCl, 1% (wt/vol) bovine serum albumin (Sigma Aldrich, Saint-Quentin Fallavier, France), 5% (vol/vol) heat-inactivated fetal bovine serum (Gibco-Invitrogen, Cergy Pontoise, France), and 0.2% (vol/vol) Tween-20 (Sigma-Aldrich). Tests were performed in 96-well flat-bottom chimney plates (Greiner bio one, Frickenhausen, Germany). Fifty microliters of bead mixture were added to each well. Preliminary experiments on different plasma dilutions (1/50-1/1,000) and different incubation times and temperatures showed that the dilution 1/200 and +4°C overnight incubation gave the best signal to noise ratio. Liquid was aspirated with an automatic plate washer (BioTek 405TS Microplate washer) and wells were then incubated with 100 μl of plasma (diluted 1/200 in assay buffer) for 16h at 4°C in the dark on a plate shaker at 300 rpm/min. After 3 washings with 100 μl of assay buffer, 50 μl of biotin-labeled anti-human IgG was added (BD-Pharmingen, Le Pont De Claix, France) at a concentration of 4 μg/ml in each well and incubated for 30 min in the dark while shaking at 300 rpm. Plates were washed 3 times as above, and 50 μl of streptavidin-R-phycoerythrin (Fisher Scientific/Life Technologies, Illkirch, France) at 1 μg/ml were added per well and incubated for ten min with shaking at 300 rpm. As in previous studies from our group and others, anti-human IgG was also used for NHPs [21,25]. Antigen-antibody reactions were then read on BioPlex-200 equipment (Bio-Rad, Marnes-la-Coquette. France). At least 100 events were read for each bead set, and the results were expressed as median fluorescence intensity (MFI) per 100 beads. To detect IgG antibodies to arboviruses in fecal samples, RNA-later-precipitated immunoglobulins were first resolubilized by diluting the fecal/RNA-later mixture (2 mL) with PBS–0.05% Tween 20 (7 mL), followed by incubation for 1 hour at 60°C, centrifugation (3900g for 10 minutes) to clarify the solution, and dialysis against PBS overnight at 4°C under a continuous stirring. The reconstituted extracts were then tested in the Luminex (diluted three volumes of dialysate for one volume of buffer) as previously described [24].

#### Calculation of cut-off, sensitivity, specificity and accuracy

For the samples of the panel, we used receiver operating characteristics (ROC) curve analysis to determine the cut-off values for each antigen, its sensitivity, specificity and accuracy which corresponds to the area under the curve (AUC). The ROC curve analysis was performed with the Life module of XLSTAT (Addinsoft, Paris, France) implemented in Microsoft Excel. We also determined the sensitivity, specificity and accuracy by calculating the mean MFI of negative controls for each antigen, the standard deviation to the mean (SD) and using as cut-off the mean plus three times the SD because for ONNV and USUV, no or only limited positive control samples were available. We used the Wilson method [26] to calculate online the 95% confidence intervals (CI) around the proportions (http://ww3.ac-poitiers.fr/math/prof/resso/cali/ic_phrek.html).

In the absence of positive controls for NHPs samples, we analyzed the data obtained from plasma and DBS samples with different statistical methods to determine MFI cut-off values for each antigen as reported in our previous studies on ebolavirus in NHPs [27]. We used a change-point analysis with the R package “changepoint” and calculated one single shift in the arithmetic mean with the AMOC (at most one change) method. We also fitted univariate distributions to our data and defined the cut-off based on a 0.05 risk of error [28]. The set of candidate distributions was reduced with a bootstrapped skewness-kurtosis analysis [29]. Maximum likelihood estimation was performed to select the best-fit distribution based on AIC (Akaike information criterion) using the R library “fitdistrplus” [30]. The best-fit distributions were negative binomial and negative exponential distributions and both were considered in data analyses. Data were bootstrapped 10,000 times and averaged for each antigen. Analyses were done with R software version 3.3.6. We then compared the cut-off values identified by the 3 different methods and calculated their mean as a consensus cut-off that we used in this study **(S4 Table).** We calculated separately cut-off values for samples collected as DBS (samples from the DRC) and those collected as blood in EDTA tubes (samples from Cameroon) because of the wide disparity of blood quantity collected with DBS. We considered a sample antigen reactive if MFI was above the cut-off value. Likewise, for samples collected as feces, we calculate MFI cut-off values separately on the data generate from the dialysates **(S5 Table**). We considered samples positive for a given antigen if they presented MFI above the cut-off value for this antigen.

## Results

### Performance of the arbovirus Luminex assay on a reference panel of human samples

Before proceeding to the screening of NHPs samples, we first evaluated the performance of the novel Luminex-based serological assay on the panel of human samples with known status (**S1 Table)**. To determine the assay performances, we first calculated cut-off values by two methods for 15 of the 17 antigens included. For USUV and ONNV, it was not possible to use ROC analysis because of the absence of positive controls and cut-off values were determined as 3XSD of negative samples. Results of cut-off determinations are summarized in **Table 1**. Regardless of the cut-off method used to determine the sensitivity, specificity and accuracy of each antigen, the specificity is in general high, > 95%, for the majority of the antigens. The sensitivity, on the contrary, depended on the virus and on the recombinant protein used. The sensitivity of NS1 recombinant proteins as determined by the ROC analysis method was 100% for WNV, DENV-2, DENV-3, ZIKV and >95% for DENV-1; 87% for DENV-4 and only 44% for YFV proteins. We also observed a 100% sensitivity for WNV-DIII protein. Sensitivity of CHIKV-E2 envelope protein was also high, >95% by ROC analysis. Of note, DIII recombinant proteins from the envelope, except WNV-DIII, presented weaker sensitivity, compared to NS1 recombinant proteins.

**Table 1:** Cut-off, Sensitivity, Specificity and Accuracy of antibody detection to the 17 different arbovirus recombinant proteins on the reference panel of 161 human samples.

Antigens	Cut-off calculation method	Cut-off value (MFI^a^)	N tested/ N negative	Specificity (%)	95% CI^b^	N tested/ N negative	Sensitivity (%)	95% CI	Accuracy (%)	95% CI
CHIKV_E2	Mean+3SD	324	66/65	98.48	91.0–99.0	27/24	88.89	71.0–96.0	96	92.0–97.0
	ROC	229	66/63	95.45	87.0–98.0	27/26	96.3	40.0–75.0	96	92.0–97.0
CHIKV_NSP	Mean+3SD	395	66/65	98.48	0.91–99.0	27/12	44.44	27.0–62.0	83	75.0–84.0
	ROC	246	66/65	98.48	0.91–99.0	27/20	74.07	55.0–86.0	91	86.0–93.0
ONNV_E2	Mean+3SD	362	66/64	96.97	0.91–99.0	NA/NA	NA	NA	NA	NA
ZIKV_DIII	Mean+3SD	485	66/65	98.48	0.91–99.0	16/0	0	0.0–19.0	79	70.0–80.0
	ROC	145	66/59	89.39	79.0–94.0	16/02	12.5	03.0–36.0	74	65.0–75.0
ZIKV_NS1	Mean+3SD	74	66/65	98.48	91.0–99.0	16/16	100	80.0–100	0.99	96.0–100
	ROC	345	66/66	100	94.0–100	16/16	100	80.0–100	100	100–100
YFV_NS1	Mean+3SD	421	66/65	98.48	91.0–99.0	18/5	27.78	12.0–50.0	83	75.0–85.0
	ROC	176	66/62	93.94	85.0–97.0	18/8	44.44	24.0–66.0	83	75.0–85.0
DENV1_DIII	Mean+3SD	134	66/66	100	94.0–100	23/17	73.91	53.0–87.0	93	88.0–95.0
	ROC	132	66/66	100	94.0–100	23/18	78.26	58.0–91.0	94	90.0–96.0
DENV2_DIII	Mean+3SD	842	66/65	98.48	91.0–99.0	23/4	17.39	06.0–37.0	78	69.0–78.0
	ROC	631	66/63	95.45	87.0–98.0	23/8	34.78	18.0–55.0	80	71.0–81.0
DENV3_DIII	Mean+3SD	197	66/63	95.45	87.0–98.0	23/17	73.91	53.0–87.0	90	84.0–91.0
	ROC	79	66/57	86.36	76.0.-92.0	23/21	91.3	76.0–97.0	88	81.0–89.0
DENV4_DIII	Mean+3SD	544	66/64	96.97	91.0–99.0	23/6	26.09	12.0–46.0	79	70.0–80.0
	ROC	112	66/53	80.3	69.0–88.0	23/18	78.26	58.0–91.0	80	71.0–81.0
DENV1_NS1	Mean+3SD	673	66/65	98.48	91.0–99.0	23/21	91.3	73.0–97.0	97	93.0–98.0
	ROC	550	66/64	96.97	91.0–99.0	23/22	95.65	79.0–99.0	97	93.0–98.0
DENV2_NS1	Mean+3SD	812	66/64	96.97	91.0–99.0	23/23	100	85.0–100	98	95.0–99.0
	ROC	996	66/66	100	94.0–100	23/23	100	85.0–100	100	100–100
DENV3_NS1	Mean+3SD	54	66/64	96.97	91.0–99.0	23/23	100	85.0–100	98	95.0–99.0
	ROC	200	66/66	100	94.0–100	23/23	100	85.0–100	100	100–100
DENV4_NS1	Mean+3SD	471	66/65	98.48	91.0–99.0	23/19	82.61	62.0–93.0	94	90.0–96.0
	ROC	462	66/65	98.48	91.0–99.0	23/20	86.96	67.0–95.0	96	91.0–97.0
USUV_NS1	Mean+3SD	89	66/65	98.48	91.0–99.0	NA/NA	NA	NA	NA	NA
WNV_NS1	Mean+3SD	100	66/64	96.97	91.0–99.0	11/11	100	74.0–100	97	94.0–99.0
	ROC	1297	66/66	100	94.0–100	11/11	100	74.0–100	100	100–100
WNV_DIII	Mean+3SD	309	66/65	98.48	91.0–99.0	11/11	100	74.0–100	99	96.0–100
	ROC	817	66/66	100	94.0–100	11/11	100	74.0–100	100	100–100

(CHIKV: Chikungunya virus); (ZIKV: Zika virus); (DENV: Dengue virus); (USUV: Usutu virus); (WNV: West Nile virus); (YFV: Yellow Fever virus); (ONNV: O’Nyong Nyong virus).

^a^ MFI: median fluorescence intensity

^b^ CI: confidence interval.

One hallmark of sero-detection of antibodies to arboviruses is cross-reaction due to antigenic homology between the different viruses. For the evaluation of the level of cross-reaction on our panel of 95 positive samples, we used cutoff values obtained with the ROC analysis method (except for ONNV and USUV). **S6 Table** shows the proportion of CHIKV, ZIKV, DENV, WNV and YFV positive samples that also react present with heterologous antigens. By doing so, 14/27 (52%) CHIKV+ positive samples cross-reacted with closely related ONNV-E2, and also with others flavivirus, varying from 2/27 (7.4%) to 16/27 (60%). On the other hand, flavivirus positive samples presented no or weak cross-reactions with antigens from the alphaviruses, CHIKV and ONNV, and variable rates of cross-reactions were observed among the different flaviviruses. Unsurprisingly, DENV+ positive samples highly cross-reacted with all DENV-NS1 proteins, regardless the dengue virus serotype not allowing to differentiate among dengue serotypes. Cross reactivity was also seen between the DIII proteins of the dengue serotypes; for example, of the six DENV-1+ samples, five reacted with DENV-1, DENV-3 and DENV-4 DIII and only DENV-2 DIII antigen presented no reaction with the six DENV-1 positive samples. DENV+ samples also reacted at high proportion with NS1 antigens from ZIKV (14/23, 60%), YFV (19/23, 82.6%) and WNV (9/23, 39%). All WNV+ samples cross-reacted with NS1 antigens from USUV and at different levels (0–36%) with other arboviral antigens. Finally, YFV+ samples cross-reacted with other NS1 antigens at levels equal or superior to that of cognate YFV NS1 antigen. To summarize, cross-reactions in our control panel with the different arbovirus antigens is variable and ranged between 0% and 60%.

Overall, accuracy of the different antigens evaluated was excellent (>95%) for all NS1, except YFV-NS1. CHIKV-E2 and WNV-DIII recombinant proteins also presented high accuracy. We thus decided to use only antigens with high sensitivity and specificity (i.e. CHIKV-E2, ONNV, ZIKV-NS1, DENV1-4-NS1, WNV-NS1 and DIII, USUV-NS1) for the screening of wildlife samples.

### Seroprevalence of IgG antibodies to arboviruses in multiple monkey species from Cameroon and the DRC

Overall, 2,100 samples collected from 26 different monkey species were screened (S2A Table **and S1 Data**). Total IgG antibodies to the ten selected recombinant proteins varied between 2% to 5% (**Table 2**). We analyzed more in detail the seroprevalence of IgG antibodies to the different arbovirus antigens, stratifying by monkey species (**Table 2**). Seven of 26 species screened were IgG negative towards all the antigens tested, i.e. *red-capped mangabey* (n = 7), *Hamlyn’s monkey* (n = 6), *L’Hoest’s monkey* (n = 38), *Preuss’s monkey* (n = 1), *drill* (n = 1), *mandrills* (n = 24) and *olive baboon* (n = 16). It should be noted that for four of these seven species, less than ten samples were tested. For the remaining 19 monkey species presenting IgG reaction against at least one antigen, seroprevalence varied greatly by monkey species and virus antigen. Hence, the *blue monkey* presented the highest proportion of positive samples, 31% (16/51), against CHIKV and were only marginally positive (1/51) against ZIKV and DENV-3 NS1 recombinant proteins. The next monkey species presenting high proportions of reactive samples are black colobus with 28% (2/7) reactive against CHIKV-E2 and DENV-NS1 recombinant proteins, angolan colobus and northern talapoin with 8% (2/25) and 11% (2/18) reactive samples against DENV-NS1 and WNV-DIII antigens, respectively. Red-tailed and mona monkeys presented 12% (23/181) and 11% (1/9) reactive samples against ONNV_E2 antigen. The remaining 13 monkey species presented less than 8% of reactive samples against any of the tested antigens.

**Table 2:** Seroprevalence of IgG antibodies to Chikungunya (CHIKV), O’nyong nyong (ONNV), Zika (ZIKV), Dengue (DENV), Usutu (USUV) and West Nile (WNV) viruses stratified by monkey species.

Species	Total	CHIKV E2	ONNV E2*	ZIKV NS1	DENV1 NS1	DENV2 NS1	DENV3 NS1	DENV4 NS1	At least 1 DENV NS1	USUV_NS1*	WNV NS1*	WNV DIII*	Range (%)
Allan swamp monkey	41	- ^a^	-	1/41 (2.4)^b^	-	-	-	-	-	1/41 (2.4)	2/41 (4.8)	3/41 (7.3)	(0.0–7.3)
Agile mangabey	128	3/128 (2.3)	4/61 (6.5)	2/128 (1.5)	2/128 (1.5)	-	1/128 (0.8)	2/128 (1.5)	3/128(2.3)	1/112 (0.8)	1/112 (0.8)	2/112 (1.7)	(0.0–6.5)
Red capped mangabey	7	-	-	-	-	-	-	-	-	-	-	-	(0.0–0.0)
Angolan colobus	25	-	-	1/25 (4.0)	2/25 (8.0)	1/25 (4.0)	2/25 (8.0)	2/25 (8.0)	2/25 (8.0)	2/21 (9.5)	2/21(9.5)	-	(0.0–9.5)
Mantled guereza	34	1/34 (2.9)	1/27 (3.7)	1/34 (2.9)	1/34 (2.9)	1/34 (2.9)	1/34 (2.9)	1/34 (2.9)	1/34 (2.9)	1/34 (2.9)	1/34 (2.9)	1/34 (2.9)	(0.0–3.7)
Black colobus	7	2/7 (28.0)	nt ^c^	1/7(14)	1/7 (14)	2/7 (28.0)	1/7 (14)	1/7 (14)	2/7 (28.0)	-	-	-	(0.0–28.0)
Tsuapa red colobus	86	2/86 (2.3)	3/85 (3.5)	1/86 (1.1)	1/86 (1.1)	1/86 (1.1)	-	1/86 (1.1)	3/86 (3.4)	3/85 (3.4)	1/85 (1.1)	5/85 (5.8)	(0.0–5.8)
Red tailed monkey	234	8/234 (3.4)	23/181 (12)	11/234 (4.7)	6/234 (2.6)	6/234 (2.6)	5/234 (2.1)	7/234 (3.0)	12/234 (5.1)	8/181 (4.5)	8/181 (4.5)	13/181 (7.1)	(2.6–12.0)
Mustached monkey	504	21/504 (4.1)	7/148 (4.7)	12/504 (2.3)	7/504 (1.3)	15/504 (2.9)	6/504 (1.2)	9/504 (1.7)	20/504 (3.9)	7/369 (1.8)	5/369 (1.3)	10/369 (2.7)	(1.2–4.7)
Hamlyn’s monkey	6	-	nt	-	-	-	-	-	-	nt	nt	nt	(0.0–0.0)
l’Hoest’s monkey	38	-	nt	-	-	-	-	-	-	nt	nt	nt	(0.0–0.0)
Blue monkey	51	16/51 (31.0)	nt	1/51 (1.9)	-	-	1/51 (1.9)	-	1/51 (1.9)	nt	nt	nt	(0.0–31.0)
Mona monkey	9	-	1/9 (11)	-	-	-	-	-	-	-	-	-	(0.0–11.0)
De Brazza monkey	59	-	2/43 (4.6)	2/59 (3.3)	-	-	-	1/59 (1.7)	1/59 (1.7)	2/53 (3.7)	3/53 (5.6)	4/53 (7.5)	(0.0–7.5)
Greater spot-nosed	385	6/385 (1.5)	9/210 (4.2)	17/385 (4.4)	7/385 (1.8)	18/385 (4.7)	9/385 (2.3)	10/385 (2.6)	29/385 (7.5)	12/315 (3.8)	6/315 (1.9)	7/315 (2.2)	(1.5–7.5)
Crested mona monkey	182	2/182 (1.0)	3/77 (3.8)	6/182 (3.3)	7/182 (3.8)	8/182 (4.4)	5/182 (2.7)	5/182 (2.7)	9/182 (4.9)	3/137 (2.1)	4/137 (2.9)	2/137 (1.4)	(1.0–4.9)
Preuss monkey	1	-	-	-	-	-	-	-	-	-	-	-	(0.0–0.0)
Wolf’s monkey	71	1/71 (1.4)	2/55 (3.6)	1/71 (1.4)	2/71 (2.8)	2/71 (2.8)	3/71 (4.2)	1/71 (1.4)	5/71 (7.0)	1/55 (1.8)	1/55 (1.8)	-	(0.0–7.0)
Tantalus monkey	14	-	-	-	-	-	-	-	-	-	-	1/14 (7.1)	(0.0–7.1)
Patas monkey	16	-	-	-	-	-	-	-	-	-	-	1/16 (6.2)	(0.0–6.2)
Grey cheecked mangabey	110	5/110 (4.5)	-	7/110 (6.3)	5/110 (4.5)	5/110 (4.5)	5/110 (4.5)	8/110 (7.2)	9/110 (8.1)	2/74 (2.7)	1/74 (1.3)	3/74 (4.0)	(0.9–8.1)
Black mangabey	33	-	1/29 (3.4)	-	-	-	-	1/33 (3.0)	1/33 (3.0)	-	-	-	(0.0–3.0)
Drill	1	-	-	-	-	-	-	-	-	-	-	-	(0.0–0.0)
Mandrill	24	-	-	-	-	-	-	-	-	-	-	-	(0.0–0.0)
Northern talapoin	18	-	-	1/18 (5.5)	2/18 (11.0)	1/18 (5.5)	1/18 (5.5)	1/18 (5.5)	2/18 (11)	1/18 (5.5)	-	2/18 (11.1)	(0.0–11.0)
Olive baboon	16	-	-	-	-	-	-	-	-	-	-	-	(0.0–0.0)
**Total**	**2100**	**67/2100 (3.2)**	**56/1109 (5.0)**	**65/2100 (3.0)**	**43/2100 (2.0)**	**60/2100 (2.8)**	**40/2100 (1.9)**	**50/2100 (2.3)**	**100/2100 (4.6)**	**44/1613 (2.7)**	**37/1613 (2.3)**	**54/1613 (3.3)**	**(1.9–4.9)**

^a^ -: no positive samples detected

^b^ n/N tested (percentages)

^c^: nt, not tested.

*The total of samples tested on ONNV, Usutu and WNV antigens are different to those tested on the other antigens for certain species.

Thereafter, IgG antibodies were stratified by collection sites and prevalences showed variation between 1.46% (WNV_NS1 in Cameroon) and 6.63% (ONNV_E2 in the DRC) **(Table 3)**. The proportions of ZIKV and DENV positive samples were in general higher in samples collected in Cameroon than in DRC, while IgG antibodies against CHIKV, ONNV, USUV and WNV antigens were higher in samples collected in DRC than in Cameroon **(Table 3)**. For DENV-2 and WNV, the difference was statistically significant (p = 0.0321 for DENV-2, 0.0007 and 0.0039 for WNV-NS1 and WNV-DIII, respectively). The proportion of positive samples is also unevenly distributed among the sampling sites within countries. A clear difference was observed between samples collected from pets and those obtained from feral monkeys. Hence 8/175 (4.5%) samples from pets, all from infant or juvenile monkeys living in urban areas, reacted with at least one of the antigens, while 5.2–46.6% of samples reacted with at least one antigen in samples collected from feral monkeys (>90% adults). IgG antibodies to CHIKV-E2 protein were detected at high proportion (35%) in WK, eastern DRC and in lesser extent, in GM also in eastern DRC and in EB, EW and BP in Cameroon.

**Table 3:** Seroprevalence of IgG antibodies to Chikungunya (CHIKV), O’nyong nyong (ONNV), Zika (ZIKV), Dengue (DENV), Usutu (USUV) and West Nile (WNV) viruses in monkeys stratified by collection site shown in Fig 1.

	CHIKV_E2	ONNV_E2^#^	ZIKV_NS1	DENV1_NS1	DENV2_NS1	DENV3_NS1	DENV4_NS1	> 1 DENV NS1	USUV_NS1*	WNV_NS1*	WNV_DIII*	Total^a^ (%)
*Cameroon (n = 1470)*												
Pets (n = 175)	-^b^	2 (1.1%) ^c^	-	2 (1.1)	-	1 (0.5)	1 (0.5)	2 (1.1)	-	1 (0.5)	5 (2.8)	8/175 (4.5)
BP (n = 19)	1 (5.2)	1 (5.2%)	1 (5.2)	1 (5.2)	1 (5.2)	-	1 (5.2)	1 (5.2)	1 (5.2)	-	-	3/19 (15.7)
ND (n = 65)	-	1 (1.5%)	1 (1.5)	1 (1.5)	1 (1.5)	1 (1.5)	2 (3.0)	1 (5.2)	1 (1.5)	1 (1.5)	1 (1.5)	5/65 (7.69)
YD (n = 96)	-	-	2 (2.0)	1 (1.0)	1 (1.0)	-	3 (3.1)	2 (2.0)	1 (1.0)	1 (1.0)	-	5/96 (5.20)
BQ (n = 51)	1 (1.9)	4 (7.8%)	2 (3.9)	3 (5.9)	5 (9.8)	3 (5.9)	5 (9.8)	4 (4.2)	2 (3.9)	4 (7.8)	-	13/51 (25.4)
EW (n = 228)	12 (5.2)	nt^$^	10 (4.4)	5 (2.1)	14 (6.1)	6 (2.6)	5 (2.1)	8 (3.5)	4 (2.9)*	3 (1.72)*	10 (5.7)*	44/228(19.2)
EB (n = 236)	11 (4.66)	nt	7 (3.0)	3 (1.2)	5 (2.1)	3 (1.2)	4 (1.7)	4 (4.5)	2 (1.6)*	1 (0.6)*	3 (1.8)*	25/236(10.5)
MS (n = 94)	3 (3.2)	6 (6.4%)	7 (7.4)	4 (4.2)	6 (6.4)	4 (4.2)	4 (4.2)	4 (4.5)	6 (6.4)	5 (5.4)	1 (1.0)	17/94 (18.0)
MN (n = 506)	12 (2.3)	12 (7.6%)^#^	17 (3.3)	12 (2.3)	17 (3.3)	10 (1.9)	12 (2.3)	14 (2.8)	9 (2.7)*	1 (0.3)*	9 (2.7)*	68/506(13.4)
**Total**	**40 (2.72)**	**26 (3.95)**	**47 (3.19)**	**32 (2.17)**	**50 (3.40)**	**28 (1.90)**	**37 (2.51)**	**40 (2.72)**	**26 (2.23)**	**17 (1.46)**	**29 (2.49)**	
*DRC (n = 630)*												
MBk(n = 125)	2 (1.6)	6 (4.8%)	3 (2.4)	-	2 (1.6)	1 (0.8)	-	-	5 (4.0)	8 (6.4)	6 (4.8)	20/125 (16.0)
ML (n = 45)	-	12 (2.7%)	5 (11)	2 (4.4)	1 (2.2)	2 (4.4)	1 (2.2)	2 (4.4)	3 (6.6)	4 (8.8)	10 (22)	21/45 (46.6)
MK (n = 29)	-	1 (3.4%)	1 (3.4)	1 (3.4)	2 (6.8)	1 (3.4)	4 (13)	1 (3.4)	2 (6.8)	2 (6.8)	2 (6.8)	8/29 (27.5)
KL (n = 280)	5 (1.8)	11 (4.3%)^#^	7 (2.5)	7 (2.5)	5 (1.8)	6 (2.1)	8 (2.8)	6 (2.1)	8 (3.1)*	6 (2.3)*	7 (2.7)*	32/280 (11.4)
WK (n = 39)	14 (35)	nt	2 (5.1)	1 (2.5)	-	1 (2.5)	-	-	nt	nt	nt	15/39 (38.4)
GM (n = 112)	6 (5.3)	nt	-	-	-	1 (0.9)	-	-	nt	nt	nt	7/112 (6.2)
**Total**	**27 (4.28)**	**30 (6.63)**	**18 (2.85)**	**11 (1.74)**	**10 (1.58)**	**12 (1.89)**	**13 (2.06)**	**9 (1.42)**	**18 (3.96)**	**20 (4.42)**	**25 (5.53)**	

^a^ Total number of samples reactive with one or more antigens.

^b^ -: no positive samples were identified.

^c^ number of positives (percentages).

* Total numbers tested are different: Cameroon: n = 1161(EW = 174; EB = 158; MN = 329); DRC. n = 452 (KL = 253).

^#^ Total numbers tested are different: Cameroon: n = 657 (MN = 157); DRC: n = 452 (KL = 253).

$: nt = not tested.

**Fig 2:**
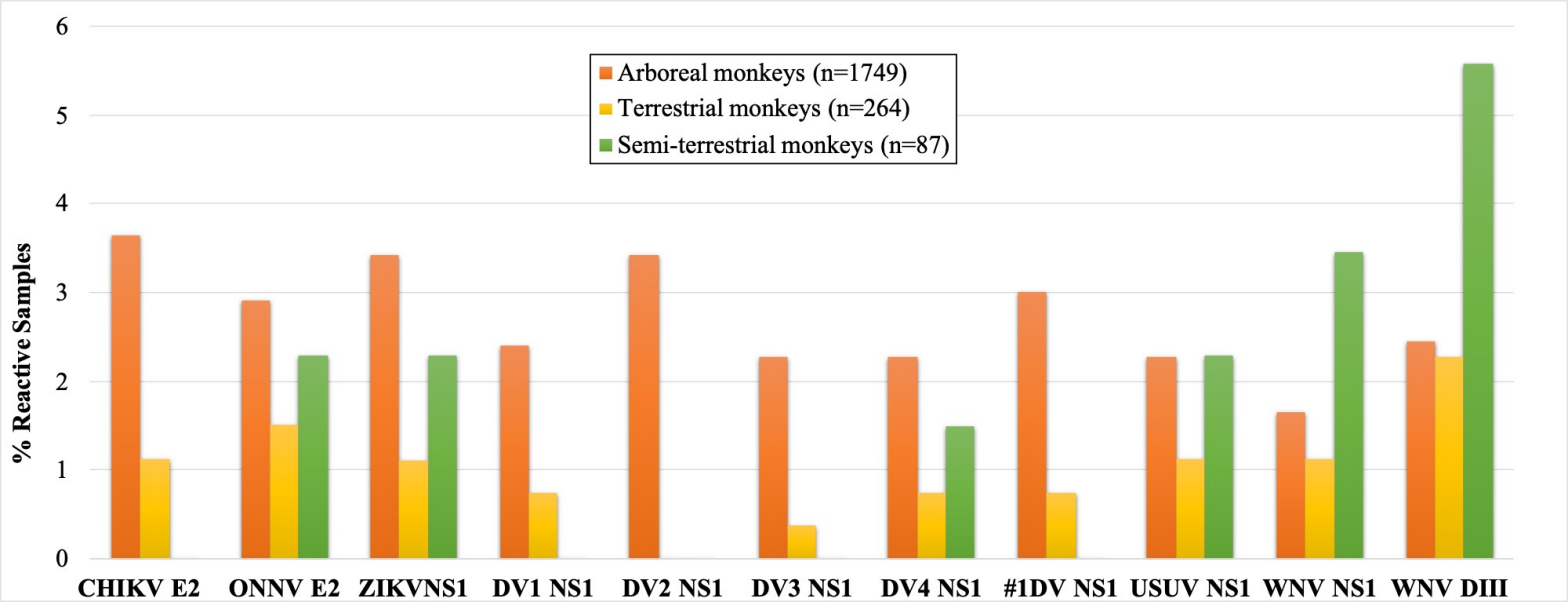
Proportions of positive samples to several arboviruses and stratified by monkey biotope/habitat. The figure shows the proportion (vertical axis) of samples positive to the different antigens reported in the horizontal axis, in the different monkey species biotopes as detailed in supplementary S7 Table.

We then stratified the proportion of positive samples by NHP biotopes and split NHPs into three groups: arboreal (n = 1,749), terrestrial (n = 264) and semi-terrestrial (n = 87) species (**Fig 2 and S7 Table).** Following this grouping, in the arboreal group positive samples were detected against all the ten antigens tested. Another remarkable observation is the quasi-absence of DENV positive samples in semi-terrestrial monkeys, with the exception of one sample (1/87) with antibodies against DENV-4 NS1 antigen. Finally, in this group of semi-terrestrial NHPs, despite the relative low number of samples tested (n = 87), high proportions of reactive samples were observed against WNV and USUV virus antigens.

We also checked for simultaneous reactions to multiple viruses (**Table 4**). For example, in Cameroon, 10% of CHIKV-E2 positive samples (n = 40), were also reactive with ONNV-E2, ZIKV-NS1 and DENV-2 NS1 antigens; 40% of USUV NS1 reactive samples (n = 22) reacted also with WNV NS1 antigen. In DRC, 18% of CHIKV-E2 reactive samples (n = 27) were reactive with ONNV-E2 and 11% with ZIKV-NS1 antigens. In both countries, DENV-NS1 reactive samples reacted with other NS1 antigens in proportions ranging between 27 and 63%. Among the USUV-NS1 reactive samples (n = 18), 72% reacted with WNV-NS1 antigen.

**Table 4:** Percentages of monkey samples reacting simultaneously with different arboviral recombinant proteins.

Cameroon	Number of reactive samples	ONNV_E2	ZIKV_NS1	DENV1_NS1	DENV2_NS1	DENV3_NS1	DENV4_NS1	USUV_NS1	WNV_NS1	WNV_DIII
CHIKV_E2	40	10.0	10.0	2.5	10.0	2.5	2.5	2.5	2.5	2.5
ONNV_E2	26		7.7	3.8	3.8	3.8	3.8	7.7	7.7	7.7
ZIKV_NS1	47			27.7	36.2	31.9	27.7	29.8	12.8	2.1
DENV1_NS1	26				69.2	50.0	80.8	23.1	19.2	3.8
DENV2_NS1	39					41.0	41.0	17.9	10.3	2.6
DENV3_NS1	22						50.0	22.7	22.7	0.0
DENV4_NS1	37							21.6	16.2	2.7
USUV_NS1	22								40.9	0.0
WNV_NS1	10									0.0
WNV_DIII	15									
DRC	Number of reactive samples	ONNV_E2	ZIKV_NS1	DENV1_NS1	DENV2_NS1	DENV3_NS1	DENV4_NS1	USUV_NS1	WNV_NS1	WNV_DIII
CHIKV_E2	27	18.5	11.1	3.7	3.7	3.7	3.7	3.7	3.7	0.0
ONNV_E2	30		13.3	6.7	10.0	3.3	10.0	13.3	16.7	23.3
ZIKV_NS1	18			22.2	16.7	27.8	27.8	33.3	33.3	11.1
DENV1_NS1	11				54.5	63.6	63.6	36.4	27.3	9.1
DENV2_NS1	10					50.0	50.0	40.0	40.0	20.0
DENV3_NS1	12						50.0	41.7	41.7	8.3
DENV4_NS1	13							30.8	30.8	7.7
USUV_NS1	18								72.2	16.7
WNV_NS1	20									10.0
WNV_DIII	25									

### Low seroprevalence of IgG antibodies to arboviruses antigens in apes from Cameroon and the DRC

African apes, including gorillas, chimpanzees and bonobos are endangered species and are on the red list of IUCN. Thus, only non-invasive sampling methods are allowed as alternative methods to study pathogens in these animals. Here we tested dialysates of 1,418 fecal samples from bonobos, chimpanzees and gorillas on the same antigens used for samples from monkeys (S1 Data). Overall, the proportions of IgG positive samples against any given arbovirus antigen was low (<5%) (**Table 5**). Gorillas, with 4.35% (35/803) reactive samples against CHIKV-E2 antigen, presented the highest proportion of IgG positive samples. There was no DENV positive sample in the 263 chimpanzees’ fecal dialysates tested. Many samples collected in the remaining 12 collection sites presented IgG antibodies against multiples arboviral antigens. For example, samples collected from gorillas in SO (Cameroon) reacted against all the ten antigens. As for monkeys, simultaneous reactions to multiple virus antigens were also observed with samples from apes. For example, 59%, 19% and 24% of CHIKV-E2 positive samples reacted also with ONNV-E2, ZIKV-NS1 and WNV-DIII antigens, respectively (**Table 6**). Likewise, DENV-1 NS1 reactive samples reacted with 33%-100% of other DENV, WNV and USUV NS1 antigens.

**Table 5:** Seroprevalence of IgG antibodies to Chikungunya (CHIKV), Zika (ZIKV), Dengue (DENV), Usutu (USUV), O’Nyong nyong (ONNV) and West Nile (WNV) viruses stratified by ape species and collection site as shown in Fig 1.

Country	Sampling site	CHIKV	ONNV	ZIKV	DV1	DV2	DV3	DV4	USUV	WNV	WNV
		E2	E2	NS1	NS1	NS1	NS1	NS1	NS1	NS1	DIII
***Bonobo***											
DRC^a^	LP (n = 14)	- ^b^	-	-	-	1 (7.14) ^c^	-	-	-	2 (14.2)	-
DRC	ML (n = 18)	-	-	1 (5.55)	-	-	-	-	-	-	-
DRC	MZ (n = 183)	1 (0.54)	-	-	-	-	-	-	-	-	-
DRC	LA (n = 137)	-	-	-	-	3 (2.1)	-	2 (1.4)	-	12 (8.75)	-
**Subtotal**	**n = 352**	**1 (0.28)**	**-**	**1 (0.28)**	**-**	**4 (1.13)**	**-**	**2 (0.56)**	**-**	**14 (3.97)**	**-**
***Chimpanzee***											
CMR	EK (n = 35)	-	2 (5.71)	1 (2.85)	-	-	-	-	-	-	-
CMR	BQ (n = 29)	-	2 (6.89)	2 (6.9)	-	-	-	-	-	2 (6.89)	-
CMR	CP (n = 5)	-	-	-	-	-	-	-	-	-	-
CMR	MB (n = 113)	1 (0.88)	-	-	-	-	-	-	-	1 (0.88)	-
DRC	BB (n = 10)	-	-	-	-	-	-	-	-	2 (20.0)	-
DRC	LS (n = 71)	-	-	-	-	-	-	-	-	-	-
**Subtotal**	**n = 263**	**1 (0.38)**	**4 (1.52)**	**3 (1.14)**	**-**	**-**	**-**	**-**	**-**	**5 (1.90)**	**-**
***Gorilla***											
DRC	IB (n = 47)	-	-	-	-	-	-	-	-	2 (4.25)	-
CMR	EK (n = 65)	-	-	-	-	-	-	-	-	-	-
CMR	BQ (n = 127)	9 (7.08)	7 (5.51)	10 (7.8)	-	-	-	1 (0.78)	-	-	3 (2.36)
CMR	SO (n = 49)	3 (6.12)	2 (4.08)	2 (4.08)	2 (4.08)	2 (4.08)	2 (4.08)	2 (4.08)	1 (2.04)	4 (8.16)	2 (4.08)
CMR	BP (n = 85)	-	-	-	-	-	-	-	-	-	-
CMR	CP (n = 195)	6 (3.07)	3 (1.53)	2 (1.02)	1 (0.51)	1 (0.51)	-	-	-	-	1 (0.51)
CMR	MS (n = 48)	-	1 (2.08)	2 (4.16)	-	-	-	-	-	-	-
CMR	DJ (n = 104)	6 (5.76)	2 (1.92)	-	-	-	-	-	-	-	-
CMR	MT (n = 40)	9 (18.3)	7 (17.5)	2 (5.00)	-	-	-	-	-	-	3 (7.5)
CMR	LB (n = 20)	-	-	-	-	-	-	-	-	-	-
CMR	MB (n = 23)	2 (8.69)	1 (4.34)	1 (4.34)	-	-	-	-	-	-	-
**Subtotal**	**n = 803**	**35 (4.35)**	**23 (2.82)**	**19 (2.36)**	**3 (0.37)**	**3 (0.37)**	**2 (0.25)**	**3 (0.37)**	**1 (0.12)**	**6 (0.74)**	**9 (1.12)**
**Total**	**n = 1418**	**37 (2.60)**	**27 (1.90)**	**23 (1.62)**	**3 (0.21)**	**7 (0.49)**	**2 (0.14)**	**5 (0.35)**	**1 (0.07)**	**25 (1.76)**	**9 (0.63)**

^a^ CMR, Cameroon; DRC, Democratic Republic of Congo

^b^ no positive samples were identified

^c^ number of positives (percentages)

**Table 6:** Percentages of ape samples reacting simultaneously with different arboviral recombinant proteins.

	Number of reactive samples	ONNV_E2	ZIKV_NS1	DENV1_NS1	DENV2_NS1	DENV3_NS1	DNV4_NS1	USUV_NS1	WNV_NS1	WNV_DIII
CHIKV_E2	37	59.5	18.9	2.7	2.7	2.7	2.7	2.7	8.1	24.3
ONNV_E2	27		23.1	3.8	3.8	3.8	3.8	3.8	11.5	34.6
ZIKV_NS1	23			13.0	13.0	8.7	8.7	4.3	8.7	17.4
DENV1_NS1	3				100	66.7	66.7	33.3	66.7	33.3
DENV2_NS1	7					28.6	57.1	14.3	85.7	14.3
DENV3_NS1	2						100	50.0	100	50.0
DENV4_NS1	5							20.0	80.0	20.0
USUV_NS1	1								100	4.5
WNV_NS1	25									8.0
WNV_DIII	9									

## Discussion

In this study, we used a novel high throughput multiplex immunoassay that included ten antigens derived from six different arboviruses, including the four Dengue serotypes, to evaluate prevalence of IgG antibodies to these viruses in a large number of samples from NHPs species from Cameroon and the DRC. Overall, we showed that IgG antibodies to arboviral antigens are low and unevenly distributed in a wide diversity of the different NHPs species and in the different sites of the two countries where the samples were collected.

### Validation of a serological screening tool

First, we developed a screening tool to detect IgG antibodies to CHIKV, ONNV, DENV, ZIKV, USUV, YFV and WNV viruses using 17 purified recombinant viral proteins. After performance evaluation, we kept only ten of these antigens for the screening of wildlife samples that presented the highest sensitivities and/or with high specificities (**Table 3**). In our assay, we found that the commonly used NS1 (DENV1-4, ZIKV, WNV) and E2 (CHIKV) antigens together with WNV-DIII recombinant proteins are the most sensitive antigens to detect IgG antibodies in our panel, with sensitivity and specificity ≥95% (**Table 1**). DIII antigens from ZIKV and DENV1-4 viruses and NSP for CHIKV were excluded because of low sensitivities. This could be explained by several parameters, including antibody kinetics, specificity restricted to one or two epitopes or recombinant protein structure impairment due to *in vitro* production issues. YFV-NS1 was not further used because of its low sensitivity, however it cannot be excluded that positive controls from vaccinated people (15/18) are not the most appropriate controls for assay development.

Sensitivity and cross-reactions are the recurrent concerns in the detection of antibodies to arbovirus infection. ELISAs, Immunofluorescence tests (IFT), multiplex microspheres assays (MIA) or Rapid Diagnostic Tests (RDT) have been used for the detection of IgG or IgM to various arbovirus [16,31]. Reported sensitivity and specificity varied greatly (39–100%) by the assay, the antibody isotype detected, the geographical environment, the time since clinical symptom onset of the infection [16]. For example, in a study performed in French Guiana in 2019 on a panel of 199 samples, including 90 ZIKV positives [32], the authors evaluated two commercially available ELISA assays and observed sensitivities of 71% and 79% and specificities of 70% and 62% for IgG detection. In our current work, the level of cross-reaction varied between 0% and 60%, depending on the antigen (**Table 3**) and correspond to what was observed in previous studies [33,34].

### Variable prevalence of IgG antibodies to arboviral antigens in monkeys from Cameroon and DRC

The overall IgG prevalence is <5% for all the six arboviruses tested in both Cameroon and the DRC. Combining all the DENV serotype NS1 antigens as one target increased IgG prevalence for some monkey species (Table 2). This strategy could thus be a valuable alternative of screening, especially if ELISA, not MIA, is to be used. This sero-prevalence to arboviruses in general is lower than in other published papers. For example, a work by Diallo and colleagues [35] reported 58.8% (10/17) prevalence of DENV in African Green Monkeys (AGMs) in Senegal using an ELISA assay for screening. In Zambia 34.4% (33/96) of samples from NHPs were positive for ZIKV using Plaque Reduction Neutralization Test (PRNT) [36]. Another study investigated the enzootic cycle of CHIKV in monkeys (*C*. *sabaeus*, *E*. *patas*, and *P*. *papio*) collected over 3 years in Senegal. They used PRNT and found 72% (479/667) seropositivity for CHIKV, and 40% of 42 randomly selected samples were also positive for ONNV [37]. Buechler et al investigated the prevalence of ZIKV exposure in wild baboons and AGMs from South Africa by ELISA assay and found that 4.9% (2/41) and 16% (4/25) had antibodies against ZIKV, respectively [38]. Another work, performed on samples collected in diverse African mammals including mandrills from Gabon, Kading [39] and colleagues used PRNT to evaluate the presence of antibodies to flaviviruses (DENV-2, WNV, YFV) and alphaviruses (CHIKV, ONNV). In the set of 25 mandrills tested, the authors found that 80% and 76% presented neutralizing antibodies to flaviviruses and alphaviruses, respectively. Proportion of neutralizing antibodies as determined PRNT, at the level of different virus was also high, ranging from 16% for ONNV to 48% for YFV. However, sample dilutions to reach 80% reduction of plaque formation are often low (1/10 to 1/100) for most of the antigens tested. In our current work, we used stringent cut-off calculation methods to determine positivity criteria and the observed prevalence could thus underestimate the actual situation in wildlife. The difference in the proportion of positive samples in mandrills from our study and the one reported by Kading and colleagues [39] in Gabon, could also be due to the location or the detection assay used (Luminex versus PRNT). While we only detect IgG antibodies, PRNT rely on the reaction of all immunoglobulin isotypes, including IgM. In other monkey species, we observed high prevalence of IgG antibodies to some antigens. For example, 31% (16/51) (**Table 2**) samples from blue monkeys, presented antibodies to CHIKV-E2 recombinant protein, 7.3% (3/41) samples from Allan swamp monkey presented IgG to WNV-DIII proteins. Variability by monkey species could be due to the ecology of the monkey species. For example, the blue monkey is an arboreal species that dwells high in the canopy of contiguous and fragmented lowland and montane tropical moist forests, riverine and gallery forests and could thus be exposed to different mosquito vectors than baboons. At higher level of classification (by biotope, **S7 Table and Fig 2**), lower prevalence was observed in terrestrial and semi-terrestrial NHPs species, probably reflecting the ecology of vectors of studied viruses. However, this can only be ascertained in conjunction with future entomological investigations. The variability we observed in the proportion of positive samples by monkey species could also be due to geography, *i*.*e*, collection site, and different ecological environment ranging from forest savannah to tropical rain forest. Age can also play a role, as can be the case for pets whose low prevalence could be rather explained by their younger age. Indeed, monkeys kept as pets are generally juveniles whose mothers have been killed by hunters in the wild. In addition, these pets were mainly living in urban areas. On the other hand, the large majority (> 90%) of bushmeat samples are from adult animals. While one would be expecting significant differences between USUV and WNV viruses and the other arboviruses because birds instead of NHP, are thought to be the reservoirs of USUV and WNV viruses, no differences were observed in the present work.

### Low prevalence of IgG antibodies to arboviruses in apes from Cameroon and the DRC

In apes, the overall IgG prevalence to the different arbovirus antigens we tested in the present work was generally lower than what was observed for monkeys. A primary possibility to explain this difference is the biological source of the sample. Hence, samples from monkeys were plasma or dried blood spots, while ape samples were feces dialysates and IgG concentration in this latter medium is lower than in plasma. Nevertheless, we showed in our earlier works on HIV/SIV and Ebola that IgG antibodies to these viruses can be detected in fecal dialysates of apes for SIV [19,23] and of survivors to Ebola virus disease, although at lower sensitivities [25]. The lower prevalence in apes can also be due to their terrestrial and semi-terrestrial behavior as observed in monkeys. In apes, we observed two distinct patterns of IgG reactivity towards arboviral antigens. In one side, in bonobos and chimpanzees, antibodies were mainly directed against WNV-NS1 antigen, and in gorillas against CHIKV-E2 antigen (**Table 5**). Significant difference for CHIKV IgG prevalence was also observed between chimpanzees and gorillas’ samples collected on the same site (MB site in Cameroon, 0.88% vs 8.69%, *p = 0*.*039*). This dichotomy could be explained by differential behaviors between the *Pan* genus and gorillas. Indeed, while gorillas spend their nights in nests on the ground, common chimpanzees and bonobos have their nests in the trees. They are thus probably exposed to different mosquito vectors. Like for monkeys, seroprevalence of IgG positive samples is also unevenly distributed in sampling sites and can thus also explain differences in species. For example, most of WNV-NS1 positive bonobos samples were from LA site. This variability by sampling site warrants further investigation, especially at the entomological level.

### Limitations of the present study

A first limitation of our study is the absence of control panel with a set of well documented samples for each of the viruses we studied here, e.g. USUV and ONNV. This could have improved the evaluation of the sensitivity of our assay. A second limitation of this novel assay is the absence of sylvatic virus antigens. Including these antigens in our Multiplex assay could have increased the chances to detect antibodies against viruses circulating in wildlife. However, DENV serotypes for which sylvatic counterparts have been identified (DENV-1,-2, and 4), amino acids identity in NS1 ranged from 91% to 97% and for the envelope, between 93% and 98% (**S8 Table**). Thus, based on these sequence identities, it is likely that sylvatic infections have been detected with our assay. Finally, for some species (*e*.*g*: drills and Preuss’s monkey) the sample size was very limited, making it impossible to estimate prevalence of arboviruses in these monkey species.

In summary, this is the first study that evaluated on a large scale the presence of antibodies to arboviruses in NHPs to evaluate their role in sylvatic cycles. We used in this work archived samples from a diverse set of NHPs from Cameroon and the DRC to investigate the circulation of different arboviruses in these wild animals. Archived samples have helped to address multiple questions relevant to the field of infectious diseases [37]. Because of the overall low IgG prevalence observed in our study, we cannot conclude that monkeys are an important wildlife reservoir for arboviruses to maintain the sylvatic viral cycle, especially given the relative low numbers of NHPs living in close proximity to humans as compared to birds, bats or rodents which are also suspected to play a role in the sylvatic life cycle for some of this viruses. Rather, NHPs might be intermediate hosts of these pathogens. Additional research is still needed in the improvement of detection tools and to elucidate the sylvatic reservoirs of arboviruses and their potential impact on human health.

## Supporting information

S1 Table Characteristics of the panel of human plasma samples used to validate the arbovirus Luminex assay. (DOCX)

S2 Table **A:** Number of monkey samples collected from different sites in Cameroon and the Democratic Republic of Congo (DRC). Location of sites are shown in Fig 1 with the same abbreviations. **B**: Number of bonobo, chimpanzee and gorilla fecal samples collected from different sites in Cameroon and the Democratic Republic of Congo (DRC). Location of sites are shown in Fig 1 with the same abbreviations.
(DOCX)

S3 Table Recombinant proteins used in the study. (DOCX)

S4 Table Cut-off values obtained with the different methods for each antigen for monkey samples collected in Cameroon (CMR) as whole blood and as dried blood spots (DBS) in the Democratic Republic of Congo (DRC). (DOCX)

S5 Table Cut-off values obtained with the different methods for each antigen on ape fecal samples. (DOCX)

S6 Table Level of cross-reactivity with recombinant antigens from other arboviruses in the reference panel of human plasma samples. (DOCX)

S7 Table Number and proportions of positive monkey samples to arboviruses and stratified by biotope/habitat. (DOCX)

S8 Table Amino acid identity matrix between diverse human and sylvatic Dengue viruses envelope and NS1 proteins. (PDF)

S1 Data Raw data used for the manuscript. (XLSX)
