# Supplementary material for: Multiplex detection of antibodies to Chikungunya, O’nyong-nyong, Zika, Dengue, West Nile and Usutu viruses in diverse non-human primate species from Cameroon and the Democratic Republic of Congo
Source: PLoS Negl Trop Dis. 2021 Jan 21;15(1):e0009028. doi: 10.1371/journal.pntd.0009028 (PMC7853492; doi:10.1371/journal.pntd.0009028)
Supplement: S1 Table — (DOCX) [file pntd.0009028.s001.docx]

**S1 Table.** Characteristics of the panel of human plasma samples used to validate the arbovirus Luminex assay.

| Sample serostatus | France | Belgium | Colombia | DRC |
| --- | --- | --- | --- | --- |
|  |  |  |  |  |
| Negative (n=66) | 61 | 5 | 0 | 0 |
| CHIKV+ (n=27) | 1 | 5 | 0 | 21 |
| DENV+ (n=23)* | 5 | 6 | 12 | 0 |
| WNV+ (n=11) | 11 | 0 | 0 | 0 |
| YFV+ (n=18) | 0 | 10 | 5 | 3 |
| ZIKV+ (n=16) | 2 | 14 | 0 | 0 |

CHIKV, Chikungunya virus ; ONNV, O’nyong nyong virus, ZIKV, Zika virus ; DENV, Dengue virus; USUV, Usutu virus; WNV, West Nile virus

*among the 23 DENV, 6 were DENV-1, 7 DENV-2, 4 DENV-3, 3 DENV-4 and 3 DENV1-4 serotypes.
