# Supplementary material for: Multiplex detection of antibodies to Chikungunya, O’nyong-nyong, Zika, Dengue, West Nile and Usutu viruses in diverse non-human primate species from Cameroon and the Democratic Republic of Congo
Source: PLoS Negl Trop Dis. 2021 Jan 21;15(1):e0009028. doi: 10.1371/journal.pntd.0009028 (PMC7853492; doi:10.1371/journal.pntd.0009028)
Supplement: S2 Table — A: Number of monkey samples collected from different sites in Cameroon and the Democratic Republic of Congo (DRC). Location of sites are shown in Fig 1 with the same abbreviations. B: Number of bonobo, chimpanzee and gorilla fecal samples collected from different sites in Cameroon and the Democratic Republic of Congo (DRC). Location of sites are shown in Fig 1 with the same abbreviations. (DOCX) [file pntd.0009028.s002.docx]

**S2A Table:** Number of monkey samples collected from different sites in Cameroon and the Democratic Republic of Congo (DRC). Location of sites are shown in Figure 1 with the same abbreviations

|  |  |  | **Cameroon** | | | | | | | | | **Democratic Republic of Congo** | | | | | |  |
| --- | --- | --- | --- | --- | --- | --- | --- | --- | --- | --- | --- | --- | --- | --- | --- | --- | --- | --- |
| **Genus** | **Species** | **Common name** | **Pets** | **BP^§^** | **ND** | **YD** | **BQ** | **EW** | **EB** | **MS** | **MN** | **MBk** | **ML** | **MK** | **KL** | **WK** | **GM** | **Total** |
|  |  |  |  |  |  |  |  |  |  |  |  |  |  |  |  |  |  |  |
| *Allenopithecus* | *nigroviridis* | Allan swamp monkey | - | - | - | - | - | - | - | - | - | 38 | - | 2 | 1 | - | - | **41** |
| *Cercocebus* | *agilis* | Agile mangabey | 11 | - | - | 1 | 9 | - | 31 | 13 | 60 | 3 | - | - | - | - | - | **128** |
|  | *torquatus* | Red capped mangabey | 2 | - | - | - | - | 5 | - | - | - | - | - | - | - | - | - | **7** |
| *Colobus* | *angolensis* | Angolan colobus | - | - | - | - | - | - | - | - | - | - | - | 4 | 21 | - | - | **25** |
|  | *guereza* | Mantled guereza | 1 | - | 15 | 8 | - | - | - | - | 8 | 1 | - | - | 1 | - | - | **34** |
|  | *satanus* | Black colobus | - | - | - | - | - | 7 | - | - | - | - | - | - | - | - | - | **7** |
| *Piliocolobus* | *tholloni* | Tsuapa red colobus | - | - | - | - | - | - | - | - | - | - | - | 2 | 84 | - | - | **86** |
| *Cercopithecus* | *ascanius* | Red tailed monkey | - | - | - | - | - | - | - | - | - | 35 | 42 | 12 | 106 | 6 | 33 | **234** |
|  | *cephus* | Mustached monkey | 27 | 6 | 9 | 16 | 24 | 108 | 158 | 14 | 142 | - | - | - | - | - | - | **504** |
|  | *hamlyni* | Hamlyn’s monkey | - | - | - | - | - | - | - | - | - | - | - | - | - | - | 6 | **6** |
|  | *lhoesti* | l’Hoest monkey | - | - | - | - | - | - | - | - | - | - | - | - | - | 6 | 32 | **38** |
|  | *mitis* | Blue monkey | - | - | - | - | - | - | - | - | - | - | - | - | - | 21 | 30 | **51** |
|  | *mona* | Mona monkey | 8 | - | - | 1 | - | - | - | - | - | - | - | - | - | - | - | **9** |
|  | *neglectus* | De Brazza monkey | 4 | - | - | 4 | 8 | 2 | 11 | - | 3 | 18 | 2 | 1 | 6 | - | - | **59** |
|  | *nictitans* | Greater spot-nosed | 41 | 8 | 21 | 51 | - | 69 | 7 | 35 | 144 | 9 | - | - | - | - | - | **385** |
|  | *pogonias* | Crested mona monkey | 5 | 1 | 10 | 14 | 10 | 21 | 27 | 17 | 76 | - | - | - | - | - | 1 | **182** |
|  | *preussi* | Preuss monkey | 1 | - | - | - | - | - | - | - | - | - | - | - | - | - | - | **1** |
|  | *wolfi* | Wolf’s monkey | - | - | - | - | - | - | - | - | - | 21 | 1 | 5 | 30 | 6 | 8 | **71** |
| *Chlorocebus* | *tantalus* | Tantalus monkey | 14 | - | - | - | - | - | - | - | - | - | - | - | - | - | - | **14** |
| *Erythrocebus* | *patas* | Patas monkey | 16 | - | - | - | - | - | - | - | - | - | - | - | - | - | - | **16** |
| *Lophocebus* | *albigena* | Grey cheecked mangabey | 6 | - | 10 | - | - | 1 | 2 | 15 | 73 | - | - | 1 | - | - | 2 | **110** |
|  | *aterrimus* | Black mangabey | - | - | - | - | - | - | - | - | - | - | - | 2 | 31 | - | - | **33** |
| *Mandrillus* | *leucophaeus* | Drill | 1 | - | - | - | - | - | - | - | - | - | - | - | - | - | - | **1** |
|  | *sphinx* | Mandrill | 15 | 1 | - | - | - | 8 | - | - | - | - | - | - | - | - | - | **24** |
| *Miopithecus* | *talapoin* | Northern talapoin | 7 | 3 | - | 1 | - | 7 | - | - | - | - | - | - | - | - | - | **18** |
| *Papio* | *anubis* | Olive baboon | 16 | - | - | - | - | - | - | - | - | - | - | - | - | - | - | **16** |
|  |  |  |  |  |  |  |  |  |  |  |  |  |  |  |  |  |  |  |
|  | **Total** |  | **175** | **19** | **65** | **96** | **51** | **228** | **236** | **94** | **506** | **125** | **45** | **29** | **280** | **39** | **112** | **2100** |

§ abbreviations of sites are as follows: BP, Bipindi; BQ, north of Dja; EB, Eboumetoum; EW, Ebolowa; GM, Goma; KL, Kole; MBk, Mbandaka; MK, Monkoto; ML, Malebo; MN, Mindourou; MS, Messok; ND, Nditam; WK, Walikale; YD,Yaoundé.

**S2B Table:** Number of bonobo, chimpanzee and gorilla fecal samples collected from different sites in

Cameroon and the Democratic Republic of Congo (DRC). Location of sites are shown in Figure 1 with the same abbreviations.

| Country | Collection site# | Bonobo | Chimpanzee | Gorilla |  | Total |
| --- | --- | --- | --- | --- | --- | --- |
|  |  | *Pan paniscus* | *Pan troglodytes troglodytes* | *Gorilla gorilla gorilla** |  |  |
| Cameroon | SO | - | - | 49 |  | 49 |
| Cameroon | BP | - | - | 85 |  | 85 |
| Cameroon | CP | - | 5 | 195 |  | 200 |
| Cameroon | MS | - | - | 48 |  | 48 |
| Cameroon | DJ | - | - | 104 |  | 104 |
| Cameroon | MT | - | - | 40 |  | 40 |
| Cameroon | LB | - | - | 20 |  | 20 |
| Cameroon | EK | - | 35 | 65 |  | 100 |
| Cameroon | BQ | - | 29 | 127 |  | 156 |
| Cameroon | MB | - | 113 | 23 |  | 136 |
| DRC | LP | 14 | - | - |  | 14 |
| DRC | ML | 18 | - | - |  | 18 |
| DRC | MZ | 183 | - | - |  | 183 |
| DRC | LA | 137 | - | - |  | 137 |
| DRC | BB | - | 10 | - |  | 10 |
| DRC | LS | - | 71 | - |  | 71 |
| DRC | IB* | - | - | 47 |  | 47 |
|  | Total | 352 | 263 | 803 |  | 1,418 |

# abbreviations of sites are as follows: BB, Bobangi; BP, Bipindi; BQ, north of Dja; CP, Campo; DJ. Djoum; EK, Ekom; IB, Ibanga;

LA, Lomako-Yokokala; LB, Lobéké; LS, Lusanga; MB, Mambelé ; ML, Malebo; MS, Messok;

MT, Mintom; MZ, Manzana; LP, Lempu; SO, Somalomo.

* Gorilla samples collected at IB in eastern DRC are from *Gorilla berengei graueri* species.
