# Supplementary material for: Multiplex detection of antibodies to Chikungunya, O’nyong-nyong, Zika, Dengue, West Nile and Usutu viruses in diverse non-human primate species from Cameroon and the Democratic Republic of Congo
Source: PLoS Negl Trop Dis. 2021 Jan 21;15(1):e0009028. doi: 10.1371/journal.pntd.0009028 (PMC7853492; doi:10.1371/journal.pntd.0009028)
Supplement: S3 Table — (DOCX) [file pntd.0009028.s003.docx]

**S3 Table.** Recombinant proteins used in the study.

| Virus | Protein | Antigen | Provider | Reference | Purity by SDP-PAGE |
| --- | --- | --- | --- | --- | --- |
|  |  |  |  |  |  |
| Chikungunya | Envelope | CHIKV_E2 | Interchim | A2YI70 | > 95% |
|  | Non Structural Protein 1234 | CHIKV_NSP | Cusabio | RPC23292 | > 90% |
| O'nyong-nyong | Envelope | ONNV_E2 | Centaur | IT-022-005Ep | > 95% |
| Zika | Envelope Domain 3 | ZIKV_DIII | Interchim | A2YHT0 | > 95% |
|  | Non Structural protein 1 | ZIKV_NS1 | Interchim | 40544-V07H | > 95% |
| Yellow Fever | Non Structural protein 1 | YFV_NS1 | Interchim | 80-1547 | > 95% |
| Dengue | Envelope Domain 3 | DENV1_DIII | Interchim | BWZ880 | > 95% |
|  | Envelope Domain 3 | DENV2_DIII | Interchim | 40471-V08Y3 | > 95% |
|  | Envelope Domain 3 | DENV3_DIII | Interchim | OPPA02169 | > 95% |
|  | Envelope Domain 3 | DENV4_DIII | Interchim | OPPA02021 | > 95% |
|  | Non Structural protein 1 | DENV1_NS1 | Biorad | PIP047A | > 95% |
|  | Non Structural protein 1 | DENV2_NS1 | Biorad | PIP048A | > 95% |
|  | Non Structural protein 1 | DENV3_NS1 | Biorad | PIP049A | > 95% |
|  | Non Structural protein 1 | DENV4_NS1 | Biorad | PIP050A | > 90% |
| Usutu | Non Structural protein 1 | USUV_NS1 | Abcam | Ab218552 | > 95% |
| West Nile | Non Structural protein 1 | WNV_NS1 | Interchim | 40346-V07H | > 95% |
|  | Envelope Domain 3 | WNV_DIII | Interchim | 40345-V08Y | > 95% |
