# Supplementary material for: Multiplex detection of antibodies to Chikungunya, O’nyong-nyong, Zika, Dengue, West Nile and Usutu viruses in diverse non-human primate species from Cameroon and the Democratic Republic of Congo
Source: PLoS Negl Trop Dis. 2021 Jan 21;15(1):e0009028. doi: 10.1371/journal.pntd.0009028 (PMC7853492; doi:10.1371/journal.pntd.0009028)
Supplement: S4 Table — (DOCX) [file pntd.0009028.s004.docx]

**S4 Table.** Cut-off values obtained with the different methods for each antigen for monkey samples collected in Cameroon (CMR) as whole blood and as dried blood spots (DBS) in the Democratic Republic of Congo (DRC).

| **Antigen** | **Country** | **Binomial** | **Exponential** | **ChangePoint** | **Mean cutoff (3 methods)** |
| --- | --- | --- | --- | --- | --- |
| **CHIKV_E2** | CMR | 7646 | 6202 | 2248 | **5365** |
|  | DRC | 4100 | 2680 | 1491 | **2757** |
| **CHIKV_NSP** | CMR | 849 | 355 | 1314 | **839** |
|  | DRC | 113 | 108 | 74 | **98** |
| **ONNV_E2** | CMR | 3691 | 1448 | 1614 | **2251** |
|  | DRC | 404 | 351 | 435 | **397** |
| **ZIKV_DIII** | CMR | 444 | 276 | 649 | **456** |
|  | DRC | 247 | 146 | 471 | **288** |
| **ZIKV_NS1** | CMR | 8857 | 4806 | 2490 | **5384** |
|  | DRC | 2463 | 1258 | 1331 | **1684** |
| **YFV_NS1** | CMR | 4558 | 2317 | 1760 | **2878** |
|  | DRC | 1143 | 601 | 719 | **821** |
| **DENV1_DIII** | CMR | 705 | 394 | 767 | **622** |
|  | DRC | 66 | 53 | 50 | **56** |
| **DENV2_DIII** | CMR | 518 | 435 | 732 | **562** |
|  | DRC | 204 | 165 | 238 | **202** |
| **DENV3_DIII** | CMR | 522 | 378 | 397 | **432** |
|  | DRC | 127 | 93 | 99 | **106** |
| **DENV4_DIII** | CMR | 1044 | 697 | 1212 | **984** |
|  | DRC | 150 | 116 | 160 | **142** |
| **DENV1_NS1** | CMR | 2343 | 1649 | 2407 | **2133** |
|  | DRC | 498 | 314 | 713 | **508** |
| **DENV2_NS1** | CMR | 4995 | 3266 | 2299 | **3520** |
|  | DRC | 1315 | 696 | 1879 | **1297** |
| **DENV3_NS1** | CMR | 911 | 463 | 1301 | **892** |
|  | DRC | 120 | 73 | 193 | **129** |
| **DENV4_NS1** | CMR | 2059 | 1428 | 2138 | **1875** |
|  | DRC | 444 | 264 | 460 | **389** |
| **USUV_NS1** | CMR | 680 | 200 | 1124 | **668** |
|  | DRC | 62 | 48 | 164 | **91** |
| **WNV_NS1** | CMR | 678 | 206 | 1432 | **772** |
|  | DRC | 46 | 35 | 77 | **53** |
| **WNV_DIII** | CMR | 506 | 223 | 566 | **432** |
|  | DRC | 50 | 50 | 44 | **48** |
