# Supplementary material for: Multiplex detection of antibodies to Chikungunya, O’nyong-nyong, Zika, Dengue, West Nile and Usutu viruses in diverse non-human primate species from Cameroon and the Democratic Republic of Congo
Source: PLoS Negl Trop Dis. 2021 Jan 21;15(1):e0009028. doi: 10.1371/journal.pntd.0009028 (PMC7853492; doi:10.1371/journal.pntd.0009028)
Supplement: S5 Table — (DOCX) [file pntd.0009028.s005.docx]

**S5 Table.** Cut-off values obtained with the different methods for each antigen on ape fecal samples.

| Antigen | Binomial | Exponential | ChangePoint | Mean of the 3 methods |
| --- | --- | --- | --- | --- |
| CHIKV_E2 | 49 | 47 | 78 | 48 |
| CHIKV_NSP | 23 | 22 | 24 | 22 |
| ONNV_E2 | 26 | 35 | 43 | 30 |
| ZIKV_DIII | 14 | 17 | 3 | 15 |
| ZIKV_NS1 | 25 | 25 | 38 | 25 |
| YFV_NS1 | 14 | 19 | 10 | 16 |
| DENV1_DIII | 13 | 14 | 2 | 13 |
| DENV2_DIII | 14 | 22 | 13 | 18 |
| DENV3_DIII | 14 | 15 | 10 | 14 |
| DENV4_DIII | 17 | 19 | 21 | 18 |
| DENV1_NS1 | 13 | 15 | 2 | 14 |
| DENV2_NS1 | 15 | 15 | 10 | 15 |
| DENV3_NS1 | 13 | 14 | 2 | 13 |
| DENV4_NS1 | 13 | 15 | 2 | 14 |
| USUV_NS1 | 13 | 16 | 2 | 14 |
| WNV_NS1 | 25 | 19 | 50 | 22 |
| WNV_DIII | 14 | 28 | 24 | 21 |
