# Supplementary material for: Multiplex detection of antibodies to Chikungunya, O’nyong-nyong, Zika, Dengue, West Nile and Usutu viruses in diverse non-human primate species from Cameroon and the Democratic Republic of Congo
Source: PLoS Negl Trop Dis. 2021 Jan 21;15(1):e0009028. doi: 10.1371/journal.pntd.0009028 (PMC7853492; doi:10.1371/journal.pntd.0009028)
Supplement: S6 Table — (DOCX) [file pntd.0009028.s006.docx]

**S6 Table.** Level of cross-reactivity with recombinant antigens from other arboviruses in the reference panel of human plasma samples

| Plasma sample status | CHIKV_E2 | CHIKV_NSP | ONNV_E2 * | ZIKV_DIII | ZIKV_NS1 | YFV_NS1 | DENV1_DIII | DENV2_DIII | DENV3_DIII | DENV4_DIII | DENV1_NS1 | DENV2_NS1 | DENV3_NS1 | DENV4_NS1 | USUV_NS1 * | WNV_NS1 | WNV_DIII |
| --- | --- | --- | --- | --- | --- | --- | --- | --- | --- | --- | --- | --- | --- | --- | --- | --- | --- |
|  |  |  |  |  |  |  |  |  |  |  |  |  |  |  |  |  |  |
| **CHIKV+ (n=27)** | 26/27 | 19/27 | 14/27 | 0/27 | 2/27 | 14/27 | 12/27 | 8/27 | 10/27 | 16/27 | 11/27 | 5/27 | 4/27 | 6/27 | 14/27 | 5/27 | 1/27 |
|  |  |  |  |  |  |  |  |  |  |  |  |  |  |  |  |  |  |
| **ZIKV+ (n=16)** | 0/16 | 0/16 | 0/16 | 2/16 | 16/16 | 7/16 | 2/16 | 0/16 | 8/16 | 1/16 | 5/16 | 4/16 | 5/16 | 5/16 | 0/16 | 2/16 | 0/16 |
|  |  |  |  |  |  |  |  |  |  |  |  |  |  |  |  |  |  |
| **DENV+ (n=23)** | 1/23 | 0/23 | 1/23 | 2/23 | 14/23 | 19/23 | 18/23 | 8/23 | 21/23 | 18/23 | 22/23 | 23/23 | 23/23 | 20/23 | 15/23 | 9/23 | 1/23 |
|  |  |  |  |  |  |  |  |  |  |  |  |  |  |  |  |  |  |
| **WNV+ (n=11)** | 0/11 | 0/11 | 0/11 | 0/11 | 1/11 | 0/11 | 2/11 | 2/11 | 1/11 | 3/11 | 3/11 | 3/11 | 2/11 | 4/11 | 11/11 | 11/11 | 11/11 |
|  |  |  |  |  |  |  |  |  |  |  |  |  |  |  |  |  |  |
| **YFV+**  **(n=18)** | 2/18 | 0/18 | 0/18 | 1/18 | 4/18 | 8/18 | 7/18 | 2/18 | 7/18 | 4/18 | 7/18 | 5/18 | 7/18 | 7/18 | 6/18 | 2/18 | 0/18 |
|  |  |  |  |  |  |  |  |  |  |  |  |  |  |  |  |  |  |
| **NEG**  **(n=66)** | 3/66 | 1/66 | 2/66 | 7/66 | 0/66 | 5/66 | 0/66 | 3/66 | 9/66 | 13/66 | 2/66 | 0/66 | 0/66 | 1/66 | 1/66 | 0/66 | 0/66 |

*Results based on cut-off determined with Mean+3xSD method instead of ROC analysis due to absence of positive control samples.

*(CHIKV:* Chikungunya virus*)*; *(ZIKV:* Zika virus*)*; *(DENV*: Dengue virus*)*; *(USUV:* Usutu virus*);* *(WNV:* West Nile virus*)*; (*YFV*: Yellow Fever virus); (*NEG*: Negative).
