# Supplementary material for: Multiplex detection of antibodies to Chikungunya, O’nyong-nyong, Zika, Dengue, West Nile and Usutu viruses in diverse non-human primate species from Cameroon and the Democratic Republic of Congo
Source: PLoS Negl Trop Dis. 2021 Jan 21;15(1):e0009028. doi: 10.1371/journal.pntd.0009028 (PMC7853492; doi:10.1371/journal.pntd.0009028)
Supplement: S7 Table — (DOCX) [file pntd.0009028.s007.docx]

**S7 Table.** Number and proportions of positive monkey samples to arboviruses and stratified by biotope/habitat.

| **Common species name** |  |  | **Number of Sample reactive to indicates antigens (%)** | | | | | | | | | | | **Total^a^** |
| --- | --- | --- | --- | --- | --- | --- | --- | --- | --- | --- | --- | --- | --- | --- |
| Arboreal (n=1749) | N | **Country** | **CHIKV E2** | **ONNV E2** | **ZIKVNS1** | **DV1 NS1** | **DV2 NS1** | **DV3 NS1** | **DV4 NS1** | **> 1 NS1 DENV*** | **USUV NS1** | **WNV NS1** | **WNV DIII** | N+ (%, range) |
| *Angolan colobus* | 25 | DRC | -^b^ | - | 1/25 (4.0) ^c^ | 2/25 (8.0) | 1/25 (4.0) | 2/25 (8.0) | 2/25 (8.0) | 2 (8.0) | 2/21 (9.5) | 2/21(9.5) | - | **3 (12.0; 4.1-29.9)** |
| *Mantled guereza* | 34 | DRC§ / CMR | 1/34 (2.9) | 1/27 (3.7) | 1/34 (2.9) | 1/34 (2.9) | 1/34 (2.9) | 1/34 (2.9) | 1/34 (2.9) | 1 (2.9) | 1/34 (2.9) | 1/34 (2.9) | 1/34 (2.9) | **3 (8.82; 12.4-40.0)** |
| *Black colobus* | 7 | CMR | 2/7 (28.0) | nt ^d^ | 1/7 (14) | 1/7 (14) | 2/7 (28.0) | 1/7 (14) | 1/7 (14) | 1 (14) | - | - | - | **3 (42.0; 15.8-74.9)** |
| *Tsuapa red colobus* | 86 | DRC | 2/86 (2.3) | 3/85 (3.5) | 1/86 (1.1) | 1/86 (1.1) | 1/86 (1.1) | - | 1/86 (1.1) | - | 3/85 (3.4) | 1/85 (1.1) | 5/85 (5.8) | **11 ( 12.7; 7.2-21.4)** |
| *Red tailed monkey* | 234 | DRC | 8/234 (3.4) | 23/181 (12) | 11/234 (4.7) | 6/234 (2.6) | 6/234 (2.6) | 5/234 (2.1) | 7/234 (3.0) | 6 (2.6) | 8/181 (4.5) | 8/181 (4.5) | 13/181 (7.1) | **49 (20.9; 16.2-26.6)** |
| *Mustached monkey* | 504 | CMR | 21/504 (4.1) | 7/148 (4.7) | 12/504 (2.3) | 7/504 (1.3) | 15/504 (2.9) | 6/504 (1.2) | 9/504 (1.7) | 11 (2.1) | 7/369 (1.8) | 5/369 (1.3) | 10/369 (2.7) | **69 (13.6; 10.9-16.9)** |
| *Blue monkey* | 51 | DRC | 16/51 (31.0) | nt | 1/51 (1.9) | - | - | 1/51 (1.9) | - | - | nt | nt | nt | **17 (33.3; 21.9-47.0)** |
| *Mona monkey* | 9 | CMR | - | 1/9 (11) | - | - | - | - | - | - | - | - | - | **1 (11.1; 1.9-43.5)** |
| *Greater spot-nosed* | 385 | DRC§ / CMR | 6/385 (1.5) | 9/210 (4.2) | 17/385 (4.4) | 7/385 (1.8) | 18/385 (4.7) | 9/385 (2.3) | 10/385 (2.6) | 11 (2.8) | 12/315 (3.8) | 6/315 (1.9) | 7/315 (2.2) | **58 (15; 11.8-18.9)** |
| *Crested mona monkey* | 182 | DRC§ / CMR | 2/182 (1.0) | 3/77 (3.8) | 6/182 (3.3) | 7/182 (3.8) | 8/182 (4.4) | 5/182 (2.7) | 5/182 (2.7) | 7 (3.8) | 3/137 (2.1) | 4/137 (2.9) | 2/137 (1.4) | **17 (9.3; 5.9-14.4)** |
| *Wolf’s monkey* | 71 | DRC | 1/71 (1.4) | 2/55 (3.6) | 1/71 (1.4) | 2/71 (2.8) | 2/71 (2.8) | 3/71 (4.2) | 1/71 (1.4) | 1 (1.4) | 1/55 (1.8) | 1/55 (1.8) | - | **8 (11.6; 8.8-25.6)** |
| *Grey cheecked mangabey* | 110 | DRC§/ CMR | 5/110 (4.5) | - | 7/110 (6.3) | 5/110 (4.5) | 5/110 (4.5) | 5/110 (4.5) | 8/110 (7.2) | 5 (4.5) | 2/74 (2.7) | 1/74 (1.3) | 3/74 (4.0) | **17 (15.4; 9.8-23.3)** |
| *Black mangabey* | 33 | DRC | - | 1/29 (3.4) | - | - | - | - | 1/33 (3.0) | - | - | - | - | **2 (6.0; 1.6-19.6)** |
| *Northern talapoin* | 18 | CMR | - | - | 1/18 (5.5) | 2/18 (11.0) | 1/18 (5.5) | 1/18 (5.5) | 1/18 (5.5) | 2 (11) | 1/18 (5.5) | - | 2/18 (11.1) | **4 (22.2; 9.0-45.2)** |
| **Total arboreal** | 1749 |  | **64 (3.6)** | **50 (2.8)** | **61 (3.5)** | **41 (2.3)** | **60 (3.4)** | **39 (2.2)** | **47 (2.7)** | **47(2.7)** | **40 (2.7)** | **29 (1.6)** | **43 (2.5)** |  |
| Terrestrial (n=264) |  |  |  |  |  |  |  |  |  |  |  |  |  |  |
| *Allan swamp monkey* | 41 | DRC | - | - | 1/41 (2.4) | - | - | - | - | - | 1/41 (2.4) | 2/41 (4.8) | 3/41 (7.3) | **7 (17.0; 8.5-31.2)** |
| *Agile mangabey* | 128 | DRC§/ CMR | 3/128 (2.3) | 4/61 (6.5) | 2/128 (1.5) | 2/128 (1.5) | - | 1/128 (0.8) | 2/128 (1.5) | 2 (1.5) | 1/112 (0.8) | 1/112 (0.8) | 2/112 (1.7) | **11 (8.5; 4.8-14.7)** |
| *l’Hoest monkey* | 38 | DRC | - | nt | - | - | - | - | - | - | nt | nt | nt | **0** |
| *Preuss monkey* | 1 | CMR | - | - | - | - | - | - | - | - | - | - | - | **0** |
| *Patas monkey* | 16 | CMR | - | - | - | - | - | - | - | - | - | - | 1/16 (6.2) | **1 (6.25; 1.1-28.3)** |
| *Mandrill* | 24 | CMR | - | - | - | - | - | - | - | - | - | - | - | **0** |
| *Olive baboon* | 16 | CMR | - | - | - | - | - | - | - | - | - | - | - | **0** |
| **Total terrestrial** | 264 |  | **3 (1.1)** | **4 (1.5)** | **3 (1.1)** | **2 (0.8)** | **0** | **1 (0.4)** | **2 (0.8)** | **2 (0.8)** | **2 (0.8)** | **3 (1.1)** | **6 (2.2)** |  |
| Semi-terrestrial (n=87) |  |  |  |  |  |  |  |  |  |  |  |  |  |  |
| *Red capped mangabey* | 7 | CMR | - | - | - | - | - | - | - | - | - | - | - | **0** |
| *Hamlyn’s monkey* | 6 | DRC | - | nt | - | - | - | - | - | - | nt | nt | nt | **0** |
| *De Brazza monkey* | 59 | DRC/CMR | - | 2/43 (4.6) | 2/59 (3.3) | - | - | - | 1/59 (1.7) | - | 2/53 (3.7) | 3/53 (5.6) | 4/53 (7.5) | **9 (15.2; 8.2-26.5)** |
| *Tantalus monkey* | 14 | CMR | - | - | - | - | - | - | - | - | - | - | 1/14 (7.1) | **1 (7.1; 1.2-31.4)** |
| *Drill* | 1 | CMR | - | - | - | - | - | - | - | - | - | - | - | **0** |
| **Total semi-terrestrial** | 87 |  | **0** | **2 (2.3)** | **2 (2.3)** | **0** | **0** | **0** | **1 (1.1)** | **0** | **2 (2.3)** | **3 (3.4)** | 5 (5.7) |  |
|  |  |  |  |  |  |  |  |  |  |  |  |  |  |  |

^a^ Total number of samples reactive with more Denv NS1antigens ; ^b^ no positive samples were identified; ^c^ number of positives (percentages); ^d^ nt, not tested

*Reactive to more than one DENV antigen

§ less than 10 samples
