# Supplementary material for: Multiplex detection of antibodies to Chikungunya, O’nyong-nyong, Zika, Dengue, West Nile and Usutu viruses in diverse non-human primate species from Cameroon and the Democratic Republic of Congo
Source: PLoS Negl Trop Dis. 2021 Jan 21;15(1):e0009028. doi: 10.1371/journal.pntd.0009028 (PMC7853492; doi:10.1371/journal.pntd.0009028)
Supplement: S8 Table — (PDF) [file pntd.0009028.s008.pdf]

Envelope (% amino acid identity)

	Sylvatic DV1	Sylvatic DV2	Sylvatic DV4
hDV1*	**97,6**	69,5	64,2
hDV2	68,5	**93,7**	64,0
hDV4	63,7	64,2	**95,7**

NS1 (% amino acid identity)

	Sylvatic DV1	Sylvatic DV2	Sylvatic DV4
hDV1	**97,0**	73,0	68,7
hDV2	73,0	**91,4**	72,0
hDV4	68,5	71,1	**93,0**

*: human Dengue Virus
